# Characterization of the complete plastid genome of *Porphyridium purpureum* strain CCMP1328

**DOI:** 10.1080/23802359.2017.1361358

**Published:** 2017-07-31

**Authors:** Guiqi Bi

**Affiliations:** a Key Laboratory of Marine Genetics and Breeding (OUC), Ministry of Education, Qingdao, China;; b College of Marine Life Sciences, Ocean University of China, Qingdao, China

**Keywords:** *Porphyridium purpureum* strain CCMP1328, plastid genome, assembly

## Abstract

In this study, the complete plastid genome of *Porphyridium purpureum* strain CCMP1328 was recovered through Illumina sequencing data. This complete plastid genome of *P. purpureum* was 220,483 bp in length and contained a pair of IR regions (4604 bp). The pt genome of *P. purpureum* encoded 234 genes including 199 protein-coding genes, 29 tRNA genes, one tmRNA, and six ribosomal RNA genes in IR regions. The overall GC content of *P. purpureum* cp genome is 30.4%. By phylogenetic analysis using 18S DNA fragments through NJ method, *P. purpureum* strain CCMP1328 was grouped in the *P. purpureum* cluster without further distinction. This complete plastid genomes can be subsequently used for evolution studies of red algae and provide valuable insight into dynamic evolution of group II introns.

*Porphyridium cruentum* is a species of red alga in the family Porphyridiophyceae.

The genome sequence of *Porphyridium cruentum* strain CCMP1328 was published in 2013 (Bhattacharya et al. 2013). The organelle genome information of *P. cruentum* strain CCMP1328 is still limited, even though another two plastid genomes from *Porphyridium* species have been available. In this study, the complete plastid genome of *P. purpureum* strain CCMP1328 was recovered through Illumina sequencing data. This complete plastid genomes can be subsequently used for evolution studies of red algae and provide valuable insight into dynamic evolution of group II introns.

The sample of *P. purpureum* strain CCMP1328, collected from Eel Pond, Woods Hole, MA, USA (41.5264°N, 70.67°W), can be obtained from the National Center for Marine Algae and Microbiota, East Boothbay, ME. The raw reads sequenced by Illumina Hiseq 2000 platform were retrieved from NCBI Sequence Read Archive database under Accession number: SRR747671 and SRR747672. After reads quality filtration, the clean reads were assembled by SPAdes 3.6.1 (Bankevich et al. 2012) based on default settings. We used another plastid genome of *Porphyridium cruentum* (AP012987) (Tajima et al. 2014) as a reference sequence to align the contigs and identify gaps. To fill the gap, Price (Ruby et al. 2013) and MITObim v1.8 (Hahn et al. 2013) were applied and Bandage (Wick et al. 2015) was used to identify the borders of the IR regions. The mean sequencing coverage of this pt genome is 253X and exhibits an obvious positive relationship with GC content. The complete sequence was primarily annotated by Plann (Huang and Cronk 2015) combined with manual correction. All tRNAs were confirmed using the tRNAscan-SE search server (Lowe et al. 1997). Other protein coding genes were verified by BLAST search on the NCBI website (http://blast.ncbi.nlm.nih.gov/), and manual correction for start and stop codons was conducted. The circular plastid genome map was drawn using OrganellarGenomeDRAW (Lohse et al. 2007). This complete plastid genome sequence together with gene annotations were submitted to GenBank under the accession numbers of MF401423.

The plastid genome of *Porphyridium cruentum* strain CCMP1328, with a length of 220,483 bp, has an unusual quadripartite structure compared with other red algae. The whole pt genome contains a two inverted repeat (IR) regions of 4604 bp, in which encodes two rRNA operons. The pt genome possesses 234 genes, including 199 protein coding genes, eight ribosomal RNA genes (four rRNA species), and 29 tRNA genes and one tmRNA. The overall GC content of the pt genome is 30.4%. The genome structure, gene order, GC content are similar to another pt genome of *Porphyridium cruentum* (AP012987), but the number of introns and its locations were different. Significantly, both the number of introns and genome synteny were diverse when comparisons between the two pt genomes of *Porphyridium cruentum* and *Porphyridium sordidum* (NC_031175) (Lee et al. 2016) were made.

For phylogenetic analysis assessing the relationship of this species, we selected 18S fragments from 12 *Porphyridium* species as DNA maker due to the lack of enough *Porphyridium* pt genomes. The sequence alignment was done by MUSCLE aligner (Edgar 2004) in MEGA 6 (Tamura et al. 2013) and the tree was constructed by NJ method under Maximum Composite Likelihood method with 1000 bootstrap calculations. As expected, the NJ tree exhibited three unique clusters, but *P. purpureum* strain CCMP1328 was grouped in the *P. purpureum* cluster without further distinction, caused by the shortness of 18S fragment (Figure 1).

**Figure 1.**
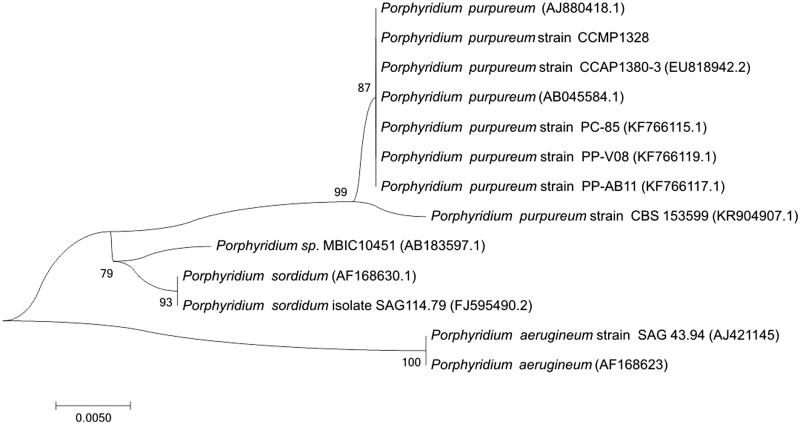
Phylogenetic tree inferred by 12 DNA sequences of 18S fragments.
